# Story time turbocharger? Child engagement during shared reading and cerebellar activation and connectivity in preschool-age children listening to stories

**DOI:** 10.1371/journal.pone.0177398

**Published:** 2017-05-31

**Authors:** John S. Hutton, Kieran Phelan, Tzipi Horowitz-Kraus, Jonathan Dudley, Mekibib Altaye, Thomas DeWitt, Scott K. Holland

**Affiliations:** 1 Division of General and Community Pediatrics, Cincinnati Children's Hospital Medical Center, Cincinnati, Ohio, United States of America; 2 Reading and Literacy Discovery Center, Cincinnati Children's Hospital Medical Center, Cincinnati, Ohio, United States of America; 3 Pediatric Neuroimaging Research Consortium, Cincinnati Children's Hospital Medical Center, Cincinnati, Ohio, United States of America; 4 Communication Sciences Research Center, Cincinnati Children's Hospital Medical Center, Cincinnati, Ohio, United States of America; 5 Education Neuroimaging Center, Faculty of Education in Science and Technology, Technion, Haifa, Israel; 6 Division of Radiology, Cincinnati Children's Hospital Medical Center, Cincinnati, Ohio, United States of America; University of Zurich, SWITZERLAND

## Abstract

Expanding behavioral and neurobiological evidence affirms benefits of shared (especially parent-child) reading on cognitive development during early childhood. However, the majority of this evidence involves factors under caregiver control, the influence of those intrinsic to the child, such as interest or engagement in reading, largely indirect or unclear. The cerebellum is increasingly recognized as playing a “smoothing” role in higher-level cognitive processing and learning, via feedback loops with language, limbic and association cortices. We utilized functional MRI to explore the relationship between child engagement during a mother-child reading observation and neural activation and connectivity during a story listening task, in a sample of 4-year old girls. Children exhibiting greater interest and engagement in the narrative showed increased activation in right-sided cerebellar association areas during the task, and greater functional connectivity between this activation cluster and language and executive function areas. Our findings suggest a potential cerebellar “boost” mechanism responsive to child engagement level that may contribute to emergent literacy development during early childhood, and synergy between caregiver and child factors during story sharing.

**Research highlights**

- Utilizes functional MRI to explore neural activation and functional connectivity during a story listening task in 4 year-old children, correlated with behaviors during a mother-child reading observation.
- Explores neurobiological correlates of shared reading behavior intrinsic to the child, whereas studies to date have largely involved parent- or home-related predictors.
- Suggests a potential neurobiological mechanism of indirect benefits of child interest and engagement in shared reading on emergent literacy skills described in behavioral studies.
- Proposes a role of cerebellar association areas in narrative processing in young children potentially influenced by level of engagement, consistent with an increasingly recognized role of the cerebellum in cognitive function and learning.

## Introduction

Illiteracy is a major public health issue disproportionately affecting low socioeconomic status (SES) populations, its annual cost estimated at over $1.2 trillion worldwide[1]. While an estimated 5–12% of reading difficulty has an organic etiology, notably dyslexia[2], the majority is attributable to inadequate resources, motivation and/or stimulation required to learn to read[1]. Recent reports have found 25% of Australian 9 year-olds scoring “below basic” for reading[3], 20% of 11 year-olds in in Britain[4], and 33 percent of US fourth graders[5], scores skewed markedly lower for low-SES and minority children[5, 6]. The seeds of environmental reading difficulty are planted early, many children arriving at school at a substantial disadvantage in readiness and increasingly unlikely to catch up with peers as academic demands accelerate[6, 7]. Thus, prevention and early intervention offer tremendous cost savings in terms of health and productivity[8–10].

As reading is an evolutionarily new, invented skill, there is no hardwired reading network in the brain. Instead, beginning in infancy, brain areas and networks adapted for other functions such as vision, language, and working memory are gradually integrated in response to reading exposure and practice.[11] This neurobiological process underlies emergent literacy, defined as “a developmental continuum between pre-reading and reading (involving) skills, knowledge, and attitudes that are precursors to reading and writing[12].” As parents are a child’s “first and most important teachers[13],” cognitive stimulation in the home, exemplified by shared reading[14–16], can greatly influence outcomes[17]. Expanding behavioral and neurobiological evidence affirms benefits of shared (especially parent-child) reading on emergent literacy skills and supporting brain networks during early childhood[18–20]. Citing this evidence base, the American Academy of Pediatrics (AAP) recommends daily shared reading beginning at birth[21], echoed by reading advocacy groups and campaigns[22]. Early interventions designed to improve outcomes often target facets of home reading environment such as access to books, frequency of reading and quality of shared (a.k.a. dialogic) reading, all largely under parent/caregiver control[23]. By contrast, while factors intrinsic to the child such as interest and engagement in shared reading are often collateral objectives assumed to provide long-term benefits, relatively little is known about their influence on emergent literacy, with largely indirect effects described[12, 24, 25] and no neurobiological studies to date. As reading engagement tends to be low in low-SES households[26], often muting benefits of shared reading[27, 28], it is particularly important to understand mechanisms by which this reciprocal process may operate in the developing brain, and their potential influence on emergent literacy in this vulnerable population.

Recent studies have found positive correlation between quantitative and qualitative aspects of home reading environment and increased activation of brain circuits supporting foundational emergent literacy skills, including semantic processing[18, 19], visual imagery[18], receptive and expressive language[19, 29], and social-emotional integration[19]. These circuits reside largely in the left cerebral cortex, within classic language, limbic and association networks[30]. While long thought to solely support motor function, mounting imaging-based evidence over the past three decades has clearly documented a major cerebellar role in cognitive abilities, notably language, executive function and social-emotional processing[31], the majority of cerebral-cerebellar connectivity involving high-level association cortex[31, 32]. Given uniform cellular architecture and contralateral, polysynaptic feedback loops, it has been proposed that the cerebellum plays a similar modulatory (“smoothing”) role for motor and cognitive skills[31, 33], facilitating rehearsal, refinement and learning[31, 34, 35]. Consistent with this view, right-sided cerebellar activation has often been cited in studies of narrative comprehension and reading[36, 37], mapping to left prefrontal and language areas. Association between cerebellar microstructure and reading component skills in school-age children has also been recently described[38]. To what degree cerebellar development and function influence emergent literacy during rapid stages of neurodevelopment prior to kindergarten[39], and their influence by shared reading behaviors, have not been studied.

Neurobiological differences during early childhood predate[40] and can predict behavioral differences, including fundamental emergent literacy skills[41–43], in turn predicting long-term outcomes[44, 45]. Thus, neuroimaging has the potential to provide mechanistic insights into traits, abilities (e.g. phonological awareness; [46]) and functional networks[47], synergistic with behavioral evidence and informing eco-bio-developmental models advocated by the AAP and US National Institutes of Health[48]. The objective of our study was to assess the relationship between child engagement during shared (mother-child) reading via direct observation and neural activation utilizing an established fMRI story listening task[49], in a sample of preschool-age children. Given indirect effects described in behavioral studies[12, 24], we hypothesized that children with higher levels of engagement would show greater activation in brain areas supporting attention (dorsal frontal-parietal) and narrative comprehension (left inferior frontal, superior temporal, inferior parietal).

## Methods

### Ethics

Of the 41 eligible mother-child dyads agreeing to participate by phone, 32 arrived for their study visit where written informed consent was obtained from mothers including a clear opt-out procedure. Families were compensated for time and travel, and our study, including the informed consent procedure and document, was approved by the Cincinnati Children’s Hospital Medical Center Institutional Review Board. Consent forms and other potentially identifying study materials were stored in a secure locked cabinet dedicated to this study.

### Participants

Our study involved 22 mother-daughter dyads recruited from a longitudinal home injury prevention trial serving low-SES mothers, based at our institution (Cincinnati Home Injury Prevention (CHIP) trial. We identified 168 girls between 3.0 and 4.5 years of age, with a goal to enroll 40 who would be 4 years old (the oldest in the CHIP cohort) during their study visit, for a target sample size of 26 accounting for a 65% MRI success rate[50]. In addition to female gender and age at scanning, inclusion criteria were: full-term gestation, right-handed, native English speakers from monolingual households, no history of head trauma with loss of consciousness or stimulant use, and no standard contraindications to MRI. We chose to sample girls exclusively due to higher MRI success rates in our desired age range (67% versus 41%[51]), and well-described gender dimorphisms negligibly influencing our story listening task at this age[52, 53]. We identified 105 girls who would be approximately 4 years old during our study window (oldest in the cohort). Of these, 55 were unable to be contacted, 5 excluded due to developmental delay, and 4 for concerns about MRI. Of the 41 agreeing to participate, 32 arrived for their visit where written informed consent was obtained as described above. Of these, 22 completed MRI and video observation (69%).

### Shared reading observation

Video observations of mother-child reading were obtained in accordance with an explicit protocol, including arrangement of the room and a script for research coordinators. Following MRI, the mother and child were directed to a private waiting room and encouraged to relax while discharge materials were being compiled. Mothers were informed that video would be in use for research purposes, though not specifically for reading assessment, a high-definition webcam unobtrusively mounted utilizing Microsoft Movie Maker software. On a table were clearly arranged: popular magazines, a table tent providing a Wi-Fi password, and a new children’s picture book (*The Little Engine That Could*, Watty Piper and Loren Long, Philomel Books 2005). If the mother or child did not spontaneously choose the book within 2 minutes, a research coordinator advised them that it was theirs to take home and to read it if they like, with no further coaching. After 15 minutes, the research coordinator entered to conclude the visit.

### Child engagement scoring

Video observation scoring was performed by the principal investigator and 2 additional scorers (1 medical student, 1 Master’s-level research coordinator) explicitly trained in dialogic reading methodology via in-person practice sessions and an online module[54], as described in Hutton et al[19]. Child engagement during shared reading was scored via a scale developed by the investigator and reviewed by experts in measure design at our institution. Scores were as follows: 0 –not engaged (persistent attempts to do something else), 1 –somewhat engaged (often tries to do something else, redirected to the story easily), 2 –very engaged (mostly focused on the story, occasional shifts), 3 –extremely engaged (focused for the entire story with almost no shifts). To differentiate them from those with no interest whatsoever in the event of maternal refusal to read, it was agreed that children expressing strong and/or sustained interest in reading should be awarded a minimum score of 1, though actual scoring was at scorer discretion. Scoring from video practice sessions was critiqued and refined until scores reached alignment. Ambiguities or concerns during actual scoring were jointly resolved, and scores were double-entered into a secure REDCap^®^ database[55]. Maternal engagement was also scored for use in a separate study, as described in Hutton, et al[19], including behaviors such as proximity during story sharing (e.g. on lap), use of child-adjusted voice (e.g. train sound effects), and encouraging the child to turn pages.

### Functional MRI acquisition during the story listening task

Details of MRI acclimatization techniques including play-based desensitization are described by Vannest, et al[51]. MRI was performed using a 3T Philips Acheiva MRI system equipped with an Avotec audiovisual system and Real Eye, eye-tracking system. MRI acquisition included a 3D anatomical brain image for registration of functional data as well as functional MRI during stimulation with a story listening task described below. For fMRI, a time-series of 165, BOLD-weighted scans covering the entire brain with 38 slices in the axial plane were continuously acquired with voxel size 3.75x3.75 x5 mm at 2-second intervals (TR = 2) during the story listening task. All children were awake and non-sedated during fMRI.

Our story listening task consists of 11 alternating blocks of control and active conditions (6 and 5 respectively), of 30 seconds duration, for a total functional scanning time of 5 minutes 30 seconds[56]. During the active condition, a series of 5 stories of 9–10 sentences each read in a female voice was presented via headphones. The stories were designed by a speech-language pathologist with vocabulary, syntax and content appropriate for preschool-age children (download: https://www.irc.cchmc.org/software/pedaudio.php), utilized in numerous published studies[57, 58]. The control condition consisted of non-speech tones in a range of frequencies simulating human speech, to control for baseline acoustic processing. No visual stimulus was presented other than a blank screen during this task.

### Functional MRI analysis

Data pre-processing was performed using FSL software (fMRI-Brain Software Library, Oxford, UK) [59]. We utilized the FEAT (fMRI Expert Analysis Tool, version 6.00) modality of FSL for our BOLD analyses[59]. Slice timing correction, spatial smoothing, intensity normalization and were all applied, followed by magnetic field map correction and registration to the structural 3D images. Following the initial pre-processing stage, functional image time series data were entered into a first-level general linear model for voxel-wise analysis to discern activation during story listening compared to tones for each child. The design matrix was comprised of hemodynamic response function-convolved paradigm timing parameters and six frame-wise motion-parameters (3 translation, 3 rotation) as covariates of no interest, to control for the effect of subject motion. A composite motion index was calculated for each subject and examined for correlations with story task timing, which was found to be very weak in all subjects (r^2^<0.09), and not correlated with child engagement scores (p>0.05). Magnitude of motion was also not problematic: average relative mean displacement was 0.50 ± 0.46 mm and the average percentage of frames with motion exceeding 2 mm was 5.9 ± 7.3%.

Contrast maps reflecting stories>tones activation for each subject were converted to Z-score maps with statistical threshold of p<0.05, applying a false discovery rate (FDR) correction for multiple voxel comparisons across the brain. These maps were entered into a second-level analysis carried out using the FLAME (FMRIB’s Local Analysis of Mixed Effects) stage 1 modality[60–62], with Z-statistic images thresholded at p = 0.01, recently demonstrated to yield conservative results[63]. Thus, we obtained a whole-brain, group mean activation map representing mean neural activation listening to stories, minus activation listening to tones (i.e. activation attributable to the story task, excluding general acoustic processing) across all 22 children.

### Linear regression with child engagement scores

A biostatistician uninvolved with video scoring performed all fMRI analyses. General linear regression was performed applying Z-score maps representing the contrast (stories>tones) for each child as the dependent variable and mean child engagement score as the predictor variable, across all 22 children. Increased power achieved via the inclusion of higher-motion children in our second level BOLD analysis (reduced Type 2 error rate) was deemed worthwhile and unlikely to introduce systemic bias or generate spurious findings (Type 1 errors), given weak correlation of subject motion with our stories task, modest magnitude of motion, and no correlation with child engagement scores. Given that our children were female and essentially the same age (4.1 +/- 0.2 years), gender and age were not considered as covariates. Household income and maternal education were considered but excluded, given lack of univariate correlation with activation for either variable. Activation maps (stories>tones) correlated with child engagement scores, along with summary statistics for size, intensity and location of activation clusters in normalized Montreal Neurological Institute (MNI) coordinate space[64] were generated, applying a threshold of p<0.05 and FDR correction for multiple comparisons across the brain.

Group-level statistical inference was carried out using FSL’s *randomise* function,[65] a nonparametric permutation test function providing robust control over false-positive results.[63] Cluster coordinates were translated into neurological cerebellar or Brodmann Areas (BA) via FSLView[59], referencing the Harvard-Oxford Cortical Structural (2mm scale) and probabilistic cerebellar atlases[66].

### Task-based functional connectivity analysis

To assess functional connectivity between brain areas correlated with child engagement scores during our story listening task and other brain areas, a biostatistician performed a *post hoc* seed-to-voxel correlation analysis utilizing the CONN toolbox for SPM[67]. Functional data were preprocessed as described above. Additionally, prior to connectivity analysis, we also applied voxelwise temporal denoising of the BOLD signal via regression of zero- and first-order derivatives of the six motion parameters, regression of the top five principle components each of the average white matter and cerebrospinal fluid BOLD signal, and 0.008 Hz high pass filtering. As connectivity analyses are highly susceptible to bias from head movement, following preprocessing, frames with composite movement >1mm or global mean intensity z-score > +/-6 were demarcated as outliers using the Artifact Detection Tool (ART) in CONN and censored from the data. Children with >10% of outlier frames were excluded from further group analysis, leaving 12 subject data sets remaining for connectivity analysis with average relative mean displacement of 0.15 ± 0.13 mm and average percentage of frames with motion exceeding 2 mm of 0.9 ± 1.7%.

To maximize our power with this relatively small data set, we took a hypothesis-driven approach, informed by strong evidence of right-sided Crus I/II connectivity with language domains[33, 68] and high overlap (over 40%) of our BOLD activation regression map with this anatomical region. Thus, we defined as our “seed,” the time-series corresponding to the average BOLD signal intersecting with right-sided Crus I/II. Fisher-transformed Pearson correlation coefficients were computed between this seed and every other voxel’s BOLD signal. Voxels positively correlated with the seed region were tested for significance at the group level using a one-tailed T-test (height p < 0.001, extent FDR-corrected p < 0.05). Significant cluster coordinates were then translated into neurological Brodmann Areas or cerebellar areas via FSLView and the Talairach Client software tool[69].

## Results

Demographic characteristics for our sample are described in Table 1.

**Table 1:** Demographic characteristics.

Characteristic		n	%
**Age (years)**	4 (4.1 +/- 0.2)	22	100
**Gender**	Female	22	100
**Annual household income ($)**	Under 5,000	10	45
	5,000–15,000	7	32
	15,000-30-000	2	9
	30,000–50,000	3	14
**Maternal Education Level**	High school graduate or less	12	55
	Some college	9	41
	College graduate	1	4

### Child engagement scores

A total of 6 mother-child dyads initiated shared reading spontaneously (27%; 3 prompted via the child and 3 via the mother), 10 read after prompting by a research coordinator (46%), and 6 did not read despite prompts (27%). Of mothers who read, 12 read the book in its entirety (75%) and 4 partially (25%). Of the 4 that stopped reading, 3 were due to the child losing interest (75%), and 1 due to the mother losing interest. Of the 6 mothers who did not read, 5 were due to maternal distraction (4 by maternal smartphone, 1 by another caregiver), 3 despite multiple entreaties from the child. Ten mothers (45%) used smartphones during the video session, 2 of them for the entire session. Children expressing strong and/or sustained interest in reading yet whose mothers did not read to them (n = 3) were awarded points based on their level of expressed interest (e.g. repeated entreaties, focused browsing of the book independently). All children received an engagement score irrespective of whether their mother agreed to read to them.

**Fig 1:**
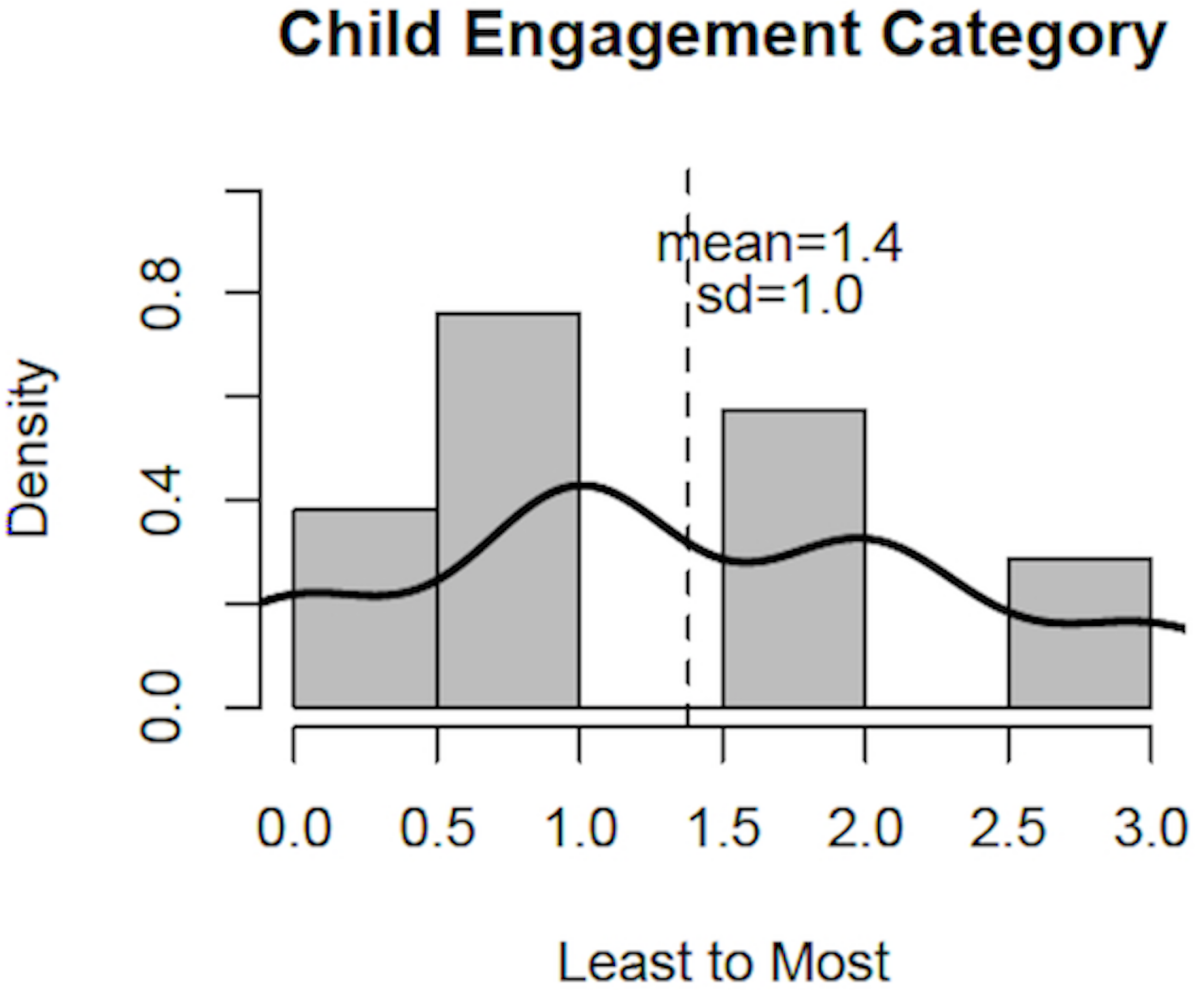
Observed child engagement scores. Histogram and density curve for observed child engagement scores, including mean (dashed line) and standard deviation (sd). Total score for each child was the mean of 3 reviewer scores.

Inter-rater reliability for child engagement scores was high (Cronbach’s alpha = 0.87). All children received an engagement score based on their interest and engagement in shared reading, irrespective of whether their mother agreed to read to them, as described above. For each child, the mean of the 3 reviewer scores was used, resulting in a mean of 1.4 (somewhat to very engaged), and standard deviation of 1.0. Significant, positive correlation was found between child engagement scores and proximity during reading, child page turning, parental use of child-adjusted voice and finishing the book, and negatively correlated with maternal smartphone use (p<0.05). A positive trend was found between child and maternal reading engagement scores (p = 0.08). A summary is shown in Table 2 and Fig 1.

**Table 2:** Observed child engagement scores and behaviors.

Item						
	n	%	Mean	Std	Min	Max
**Child observed score**	22	100	1.4	1	0	3
**Observed behaviors**						
Proximity—lap	2	13				
Proximity–side close	6	38				
Proximity–distant	8	50				
Page turning—never	9	56				
Page turning—sometimes	4	25				
Page turning—often	3	19				
Child-adjusted voice—never	5	31				
Child-adjusted voice—sometimes	11	69				
Child-adjusted voice—often	0	0				
Smartphone checking	10	45				

Summary of observed child engagement scores and frequencies of correlated behaviors (p<0.05). Total score was obtained averaging 3 independent reviewer scores (n = 22), with mean, standard deviation (std), minimum and maximum presented for total score.

### Group mean activation for the story listening task

**Fig 2:**
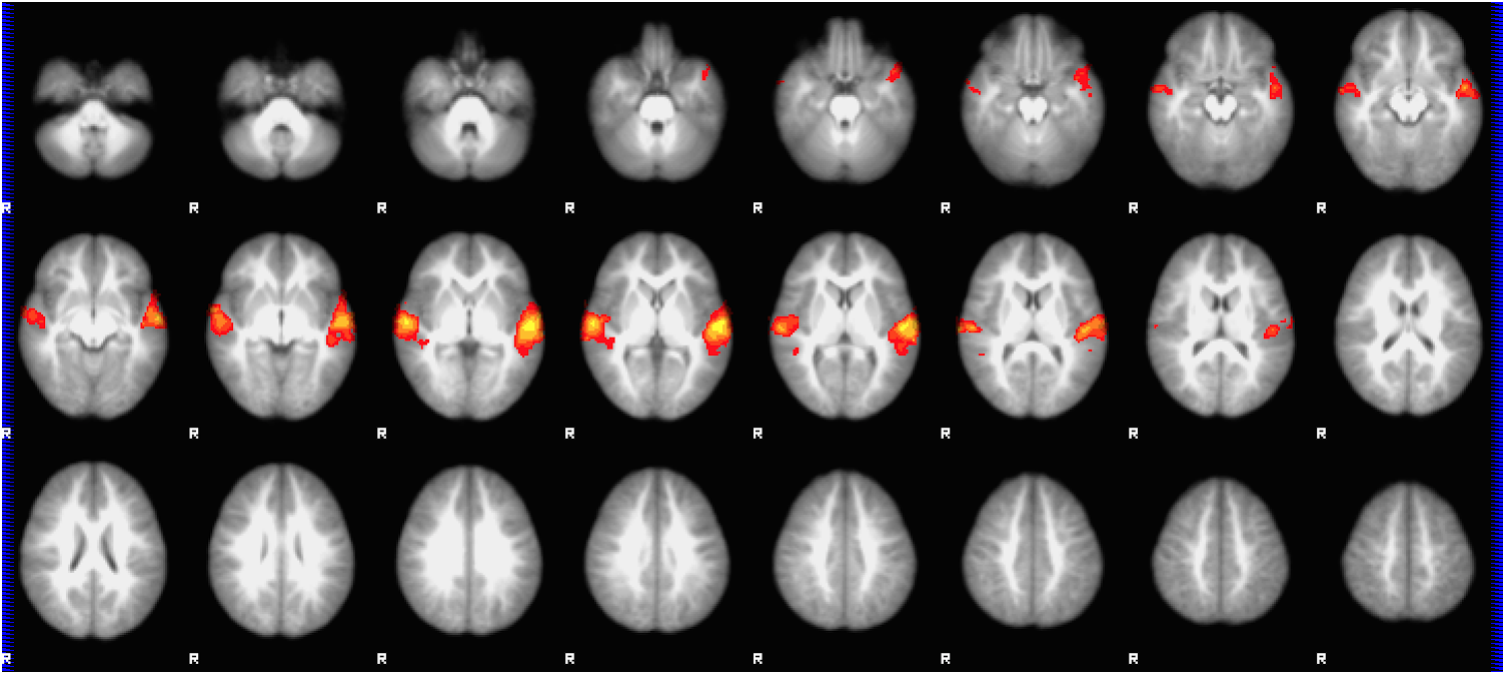
Group mean activation map for the story listening task. Group mean BOLD fMRI activation map for our story listening task (stories>tones) in 4 year-old girls (n = 22). All voxels significant at *p*<0.05 (FDR corrected), slice thickness 5 mm for contiguous slices. Slices range from *z* = -28 to *z* = 74 in MNI coordinate space. Color scale ranges from *t* = 1.25 (cooler) to 4 (hotter). Radiological orientation, left = right, right = left.

Group mean activation for the stories condition compared to baseline tones involved bilateral, left-lateralized cortical and subcortical regions involved with acoustic, phonological and semantic language processing, similar to prior studies involving this task and age range[49, 58]. Fig 2 displays the group mean activation map for the condition of stories>tones for all participants who completed the task (n = 22, display threshold highlights voxels p<0.05, FDR correction).

### Regression of neural activation with child engagement scores

**Fig 3:**
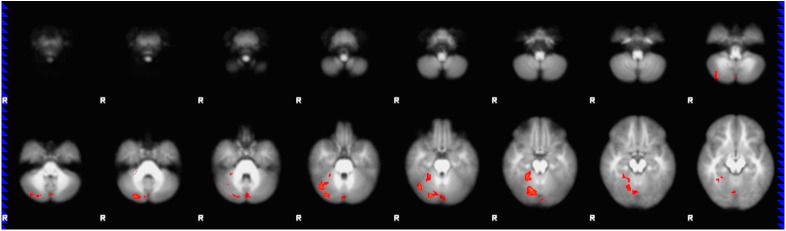
Regression map for the story listening task (stories>tones activation) with child engagement score applied as explanatory variable. Regression map for the story listening task (stories>tones; n = 22), with child engagement score as the explanatory variable. Total cluster size 1164 voxels (p<0.05, FDR corrected), with center of gravity at (x = 21, y = -67, z = -25; R posterior cerebellum) in MNI coordinate space and z-score local maxima 3.22–3.93. Shown as 5 mm slices from *z* = -64 to *z* = -12 in MNI coordinate space. Color scale from *t* = 1.25 (cooler) to 4 (hotter). Radiological orientation, left = right, right = left.

**Fig 4:**
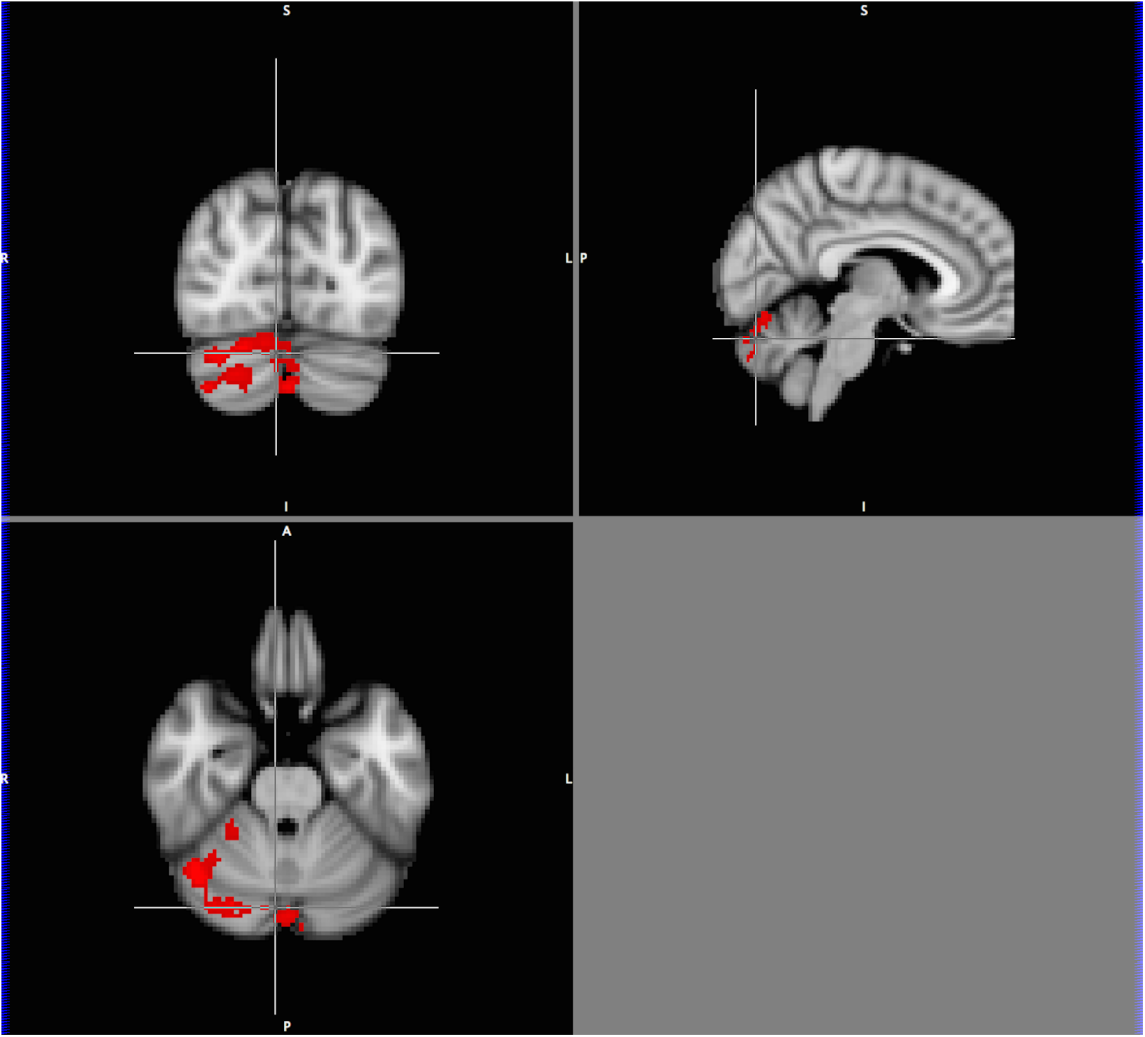
Tri-planar view of neural activation (stories>tones) for the story listening task with child engagement score as explanatory variable. Orthogonal tri-planar view (origin x = 6, y = -78, z = -26, MNI coordinate space; right Crus II/Vermis VI) of cerebellar activation for the story listening task (stories>tones; n = 22), with child engagement score as explanatory variable. Total cluster size 1164 voxels (p<0.05, FDR corrected). Color scale *t* = 1.25 (cooler) to 4 (hotter). Radiological orientation, left = right, right = left, sagittal plane viewed from the right.

Child engagement scores were positively correlated with higher BOLD activation in right-sided, largely posterior cerebellar areas including (in anatomically descending order) lobule V, generally ascribed a sensorimotor role including with eye movement, extensive activation in lobule VI, Crus I and Crus II (cognitive-association areas), and smaller activation in Vermis VI and bilateral Vermis II, whose ascribed roles include emotional/affective processing[32, 70]. Less extensive, focal activation in right-sided, inferior cerebral areas was also found, involving temporal-occipital fusiform and lingual gyri, supporting higher-order visual processing, semantic association and imagery[71]. These areas are shown in Fig 3 (voxels p<0.05, FDR correction, n = 22). Fig 4 provides orthogonal sagittal, coronal and axial views (origin x = 6, y = -78, z = -26, MNI coordinate space) to more clearly illustrate the anatomical extent of activation clusters.

### Functional connectivity

**Fig 5:**
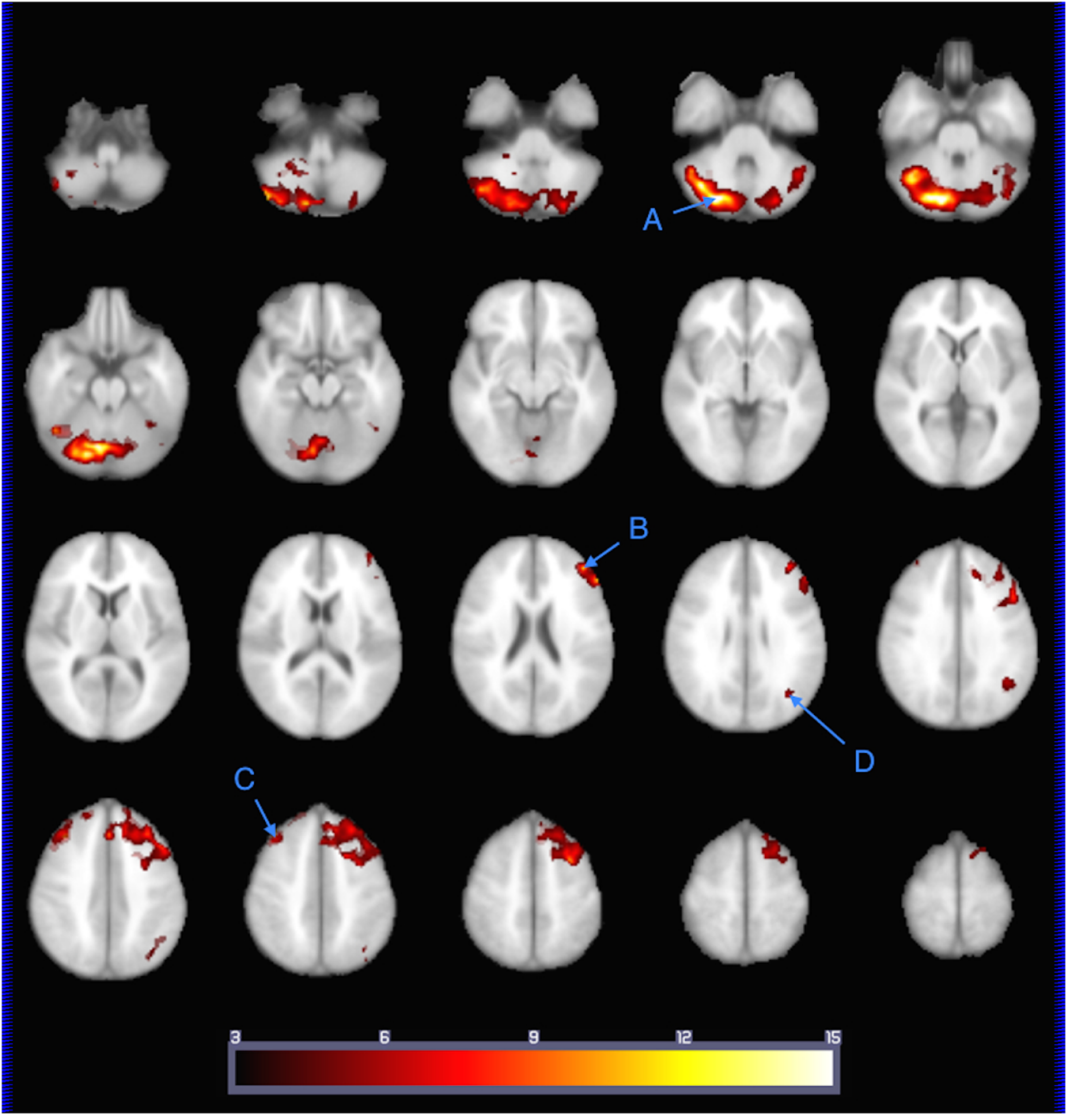
Functional connectivity map showing brain areas functionally connected with the seed activation cluster correlated with child engagement scores. Seed-to-voxel map of brain areas functionally connected with the seed activation cluster (right Crus I/II) correlated with observed child engagement scores during the story listening task (stories>tones; n = 12). Clusters at MNI coordinates: A) (x = 26, y = -78, z = -36; bilateral crus I/II and right cerebellar lobules VI and VII/VIII), B) (x = -42, y = 42, z = 22; left BA 6, 8, 9), C) (x = 38, y = 30, z = 42; right BA 8), and D) (x = -36, y = -66, z = 26; left BA 39). Color scale *t* = 3 (cooler) to 15 (hotter). Radiological orientation, left = right, right = left.

*Post hoc* seed-to-voxel functional connectivity mapping was performed in the subset of n = 12 participants whose fMRI data met the more stringent motion limitations imposed for this analysis. Significant positive correlations (one-tailed, height p<0.001, extent FDR-corrected p<0.05) between the seed region in right Crus I/II identified in our BOLD activation analysis was discovered in four discrete clusters located in: 1) right cerebellar lobules VI-VIII spanning into left Crus I/II, considered to support cognitive association[31, 70], 2) left dorsolateral prefrontal cortex (BAs 8, 9 and 46) supporting a variety of executive functions[72], 3) right superior frontal gyrus (BA 8; frontal eye fields), supporting top-down attention and gaze control[73], and 4) left angular gyrus (BA 39) ascribed multiple integrative roles, notably complex language processing[74, 75], as shown in Fig 5.

## Discussion

Shared reading, recommended to initiate at birth[21], is considered among the best means to provide constructive cognitive stimulation promoting healthy brain development during the critical span of early childhood[20, 39, 76]. Most behavioral and neurobiological evidence of benefits of shared reading on emergent literacy skills and supporting brain circuits involve factors within caregiver control, such as access to books, reading frequency and quality of dialogic reading[18–20, 76]. While widely assumed to provide long-lasting benefits, factors intrinsic to the child, such as interest and engagement in shared reading–later called “print motivation”[24]–are relatively under-studied, with little evidence of direct benefit. It has been suggested that such child-intrinsic factors exert indirect influence by catalyzing increased reading frequency, which in turn confers direct benefits[12, 77]. Higher interest and engagement are also thought to enhance the affective quality of shared reading, fueling gains in vocabulary and comprehension in home settings via greater focus on story content[24, 78], and gains in alphabet knowledge and decoding in school settings via greater focus on skill development[12]. Similarly, aspects of cognitive control such as attention and distraction inhibition are thought to exert indirect effects on emergent literacy skills and response to interventions via reinforcing more frequent, higher-quality shared reading[78]. Our findings suggest, albeit tentatively, a neurobiological mechanism by which greater child engagement during shared reading may directly influence–or “turbocharge”–the development of foundational emergent literacy skills, particularly comprehension, in preschool-age children.

The cerebellum has the largest number of neurons in the central nervous system[79], and undergoes rapid growth and development during childhood mirroring that of the cerebrum[80]. Until recently, its role was thought to be limited to motor processes, given the preponderance of motor deficits in lesion studies and difficulty mapping polysynaptic cerebro-cerebellar connections via traditional anatomical techniques[31]. The advent of functional neuroimaging has provided potent means to affirm a cerebellar role in higher-level cognitive processes, such as language, social-emotional processing and executive function[31, 33, 81, 82]. The majority of cerebellar connectivity is with cerebral association cortices, in accordance with consistent rules such as functional asymmetry, homotopicity (geometric correspondence) and contralaterality[31]. This mapping is localized in posterior-lateral areas, notably Crus I/II and lobule VI[31, 32], each highly active in our analyses. For language, strong correlation has been found between activation in Crus I/II and contralateral language areas in the dominant (usually left) cerebral hemisphere, consistent with increased functional connectivity between our seed (right Crus I/II) and left inferior frontal and angular gyri[68]. Right-sided cerebellar activation has been well described in imaging-based studies of narrative comprehension and reading[36, 37, 83, 84], consistent with the primacy of left-sided language and semantic areas in mature reading networks, and our findings suggest a cerebellar role in emergent literacy prior to kindergarten.

Given its consistent cellular architecture and polysynaptic feedback loops, it has been proposed that the cerebellum plays a similar modulatory role–a “cerebellar transform”[35]—in motor and cognitive processes[31]. Thus, as with motor planning and coordination optimizing movement, thought and emotion may be rehearsed and refined via internal models, enhancing skill development and learning[34]. Associations between cerebellar white matter microstructure and reading component skills in school-age children were recently described, consistent with this view[38]. Cerebellar activation seems to increase with greater cognitive loading, such as via novel words, stories or experiences[33], reasonably expected to be frequent during childhood and our story listening task. Increased cerebellar activation with tasks involving higher working memory demands was recently shown, including areas found in our results[85]. As with movement following lesion or surgery, learning and execution of such skills and tasks are possible without cerebellar involvement, but relatively inefficient and/or impaired, described as “dysmetria of thought”[35, 86, 87]. Our findings suggest that children who are more deeply engaged during shared reading more strongly recruit cerebellar association areas to “turbocharge” neural processing and connectivity during story listening, a potential mechanism for enhanced decoding (mediated by more robust recruitment of working memory), semantic processing, lexical access, social-emotional connection, and comprehension. This inference is consistent with the iconic image of rapt expressions on the faces of highly engaged children during preschool story time, hands shooting up to ask and answer questions. Whether such “turbocharged” activation is a consequence of constructive stimulation and nurturing, genetically determined temperament, or (most likely) multi-factorial, is beyond the scope of this paper, but merits further study.

Our findings reinforce behavioral evidence suggesting a nuanced yet broad influence of child interest and engagement in shared reading on emergent literacy skills. Contrary to our hypotheses, we did not find direct association between child engagement scores and activation in attention or semantic cerebral areas. Instead, the correlation between child engagement scores and exclusively right-sided cerebellar activation and connectivity reflect a potential enhancing (or “smoothing”[31]) effect on cerebral language, attention and association cortices. Thus, we suggest that higher child engagement during shared reading reflects fundamental differences in neural processing capacity, or efficiency, rather than simply a more favorable route to increased reading frequency[88]. It is reasonable to speculate that enhanced cognitive and social-emotional processing via this cerebellar “boost” may help make shared reading more pleasant and stimulating for the child, spurring a virtuous cycle of greater interest, inspiring caregivers to read more often with deeper emphasis on interactivity and story content, in turn fueling cerebellar activation and learning. Behavioral evidence is consistent with such a cycle, as children who view shared reading as a pleasurable experience–an “entertainment orientation”[24]–tend to manifest greater interest in reading increasingly advanced material more often[24], predicting later achievement[25]. Unfortunately, despite near-significance between maternal and child engagement scores in our video observation, many mothers exhibited low affective quality during shared reading or were frankly unresponsive to child entreaties to read, most commonly due to smartphone distraction, consistent with low reading motivation and engagement previously reported in low-SES parents[89]. As the preschool age range (3–5) is a highly sensitive period to nurture interest in reading independent of skill level and SES[90], our findings underscore the importance of interventions explicitly addressing both parent and child reading engagement, including awareness and reduction of distractions. Despite low sample size for behavioral analyses, positive correlation between child engagement scores and proximity during reading, child page turning, and use of child-adjusted voice suggests 3 relatively simple behaviors to be recommended that would likely have a positive impact on child reading engagement. Paradoxically, such approaches focused on making reading interesting, interactive and enjoyable—*fun*—tend to be more effective at promoting emergent skills than those focused on rote instruction[24, 77], and should be emphasized in anticipatory guidance and interventions.

Our study has several important strengths. Our sample of 4 year-old girls is considerably younger than most neuroimaging-based studies of emergent literacy[36, 91], with ample sample size[92] drawn from well-defined cohort, successfully applying an established fMRI story listening task paradigm[93] including connectivity-based analysis. We tested for and excluded important potential confounders, household income and maternal education. Our shared reading assessment involved observation via an explicit protocol, addressing concerns regarding reliability of parental report[94]. Our scorers were trained to apply standardized dialogic reading and child engagement criteria, with high inter-rater reliability. We applied conservative BOLD thresholding parameters, nonparametric permutation testing strictly controlling for family-wise error rate, and strict motion correction in our analyses, reducing the prospect of spurious findings[63]. The decision to include higher-motion children for increased power in our BOLD activation analysis was carefully considered and an unlikely source of bias, given weak correlation between motion parameters and our stories task, modest magnitude of motion, and non-correlation of motion with our behavioral measure. Confidence in our findings is further bolstered by the topology of the activation cluster, which aligns with current models of cerebellar function in higher-level association[31, 33], notably its role in language and reading[37, 91], which in turn aligns with behavioral evidence[12, 24]. These findings were reinforced via connectivity-based analysis, utilizing stricter motion correction and a hypothesis-driven approach. Our results build on recent evidence of positive correlation between home reading environment and maternal shared reading engagement and brain function and connectivity in preschool-age children[19, 58], suggesting synergistic neurobiological mechanisms. Together, these findings inform clinical practice during a formative stage of development via an innovative approach, reinforcing literacy promotion recommendations[21] and early reading interventions[23].

Our study also has limitations. While we attempted to contact all mothers with daughters in our target age range, we were unable to do so for many, and those who did enroll may be more engaged in their child’s development. However, by design all mother-child dyads in the CHIP cohort are at-risk for poor outcomes, limiting the range of such bias. Our decision to exclusively sample girls limits generalizability, though we view this as a strength, allowing us to efficiently collect high-quality data in young children. Furthermore, while gender dimorphisms in brain structure and function in young children have been described[56, 95], these have been found to be negligible for our story listening task[52], leading us to expect similar findings in boys. Our homogeneous sample of low-SES mothers also limits generalizability, though we see this too as a strength, as low-SES populations stand to benefit most from improved insights and interventions. A larger study involving a diverse sample would help determine to what extent SES moderates child engagement during shared reading and its influence on neural processing. Our child engagement score reflected a single snapshot, and may not be representative of longer-term behavior. However, household reading behaviors tend to be stable during the preschool period[96], and such focal observations have been reliably utilized in in assessment of home environment[97], including reading[98]. Our assessment of child reading engagement was conducted outside of the scanner, given restrictions on motion and interactivity during the scan itself. However, it is reasonable to assume that observed engagement in story reading exhibited by the child during the same study visit, reflective of longer-term experience and interest, is a fair proxy for that manifest during the story listening task. Future studies could assess child engagement during the MRI task itself via an age-appropriate post-scan questionnaire. Finally, whereas our results show compelling correlation between observed child interest in and engagement during shared reading and neural activation and connectivity during a story listening task, our study design cannot establish causation. Longitudinal studies are needed beginning in infancy, to better understand dynamic home, maternal/caregiver and child factors contributing to healthy brain development and emergent literacy.

## Conclusions

We utilized functional neuroimaging to demonstrate positive correlation between child interest and engagement in shared reading, assessed via video observation, and increased activation and functional connectivity in cerebellar association areas during a story listening fMRI task. The cerebellum is increasingly recognized to play a role in higher-level cognitive processing, typically right-lateralized for language and reading, purportedly enhancing skill rehearsal and learning. Our findings suggest an intriguing, albeit tentative, mechanism by which these factors intrinsic to the child may enhance emergent literacy development via recruitment of cerebellar association areas–a cerebellar “boost”–during story sharing, and underscore the potential of interventions targeting parent-child engagement and interest in reading.

## Supporting information

S1 File Deidentified subject data (n = 22) for children in this study utilized in analyses, including maternal education, household income, observed child engagement scores, and scores for observed mother-child reading behaviors. (XLSX)

## Abbreviations

AAP: American Academy of Pediatrics
BOLD: blood oxygen level dependent
CHIP: Cincinnati Home Injury Prevention (study)
FDR: false discovery rate
fMRI: functional magnetic resonance imaging
MNI: Montreal Neurological Institute
PTO: parietal-temporal-occipital
SES: socioeconomic status
